# Do the effects of Hormone Replacement Therapy during menopause differ by APOE genotype?

**DOI:** 10.1002/alz.095240

**Published:** 2025-01-09

**Authors:** Emily Budden, Sam Atkinson, Sam Berens, Alice Stanton, Sarah L King, Anne‐Marie Minihane, Michael Hornberger, Daniel Reisel, Louise Newson, Naji Tabet, Dorina Cadar, Claire L Lancaster

**Affiliations:** ^1^ Brighton & Sussex Medical School, Brighton United Kingdom; ^2^ University of Sussex, Brighton United Kingdom; ^3^ University of East Anglia, Norwich United Kingdom; ^4^ Newson Health, Stratford upon Avon United Kingdom; ^5^ Newson Health, Stratford‐upon‐Avon United Kingdom; ^6^ Brighton and Sussex Medical School, Brighton United Kingdom; ^7^ Centre for Dementia Studies, Brighton and Sussex Medical School, Brighton United Kingdom

## Abstract

**Background:**

Reported effects of Hormone Replacement Therapy (HRT) on late‐life neurodegenerative disease are inconsistent. Variability in the timing and formulation of HRT, plus whether an individual carries an Apolipoprotein (APOE) e4 genetic risk variant for Alzheimer’s Disease (AD), likely contribute to conflicting results. Additionally, whilst many studies have focused exclusively on the effects of exogenous oestrogen, the inclusion of testosterone in HRT appears protective against AD pathology, specifically in APOE e4 carriers. This project will investigate whether the introduction of HRT affects cognition in mid‐life, including whether benefits are influenced by HRT formulation, timing of initiation, and APOE genotype.

**Methods:**

Peri and early postmenopausal women (minimum *n* = 500*)* being newly prescribed HRT will be recruited from Newson Health specialist menopause clinics. Participants will complete a suite of online cognitive tasks targeting domains sensitive to preclinical AD (e.g., perceptual discrimination, spatial navigation), alongside questionnaires capturing cognitive, physical, and behavioural menopause symptoms, at baseline, 4‐months, and 12‐months. A tissue sample will be collected for *APOE* genotype analysis. HRT prescription and circulating blood‐hormone levels will also be available from clinic records.

**Results:**

Existing cohort data (*n* = 10,222) from Newson Health indicates 97% patients present at clinic with memory or concentration complaints. Pilot data for this study (*n =* 85, mean age 56.48 years, peri/postmenopausal women) showed HRT users (relative to non‐users) were significantly quicker on a perceptual 'odd‐one‐out’ task (*p = *.025) and trended to show better lure discrimination on an object mnemonic similarity task (*p* = .064). This project builds on this by testing whether the introduction of HRT influences cognition longitudinally, including whether effects differ by HRT formulation (estrogen/+progesterone/+testosterone), timing of initiation relative to menopause, and APOE genotype. Exploratory analysis will consider whether predicted benefits of HRT are moderated by non‐cognitive menopause symptoms, cardiovascular health, and wider lifestyle factors.

**Conclusions:**

Exploring the impact of HRT on cognition, specifically in interaction with the leading genetic risk factor for AD, will further research into how we can promote women’s brain health from mid‐life. Additionally, this research will inform best‐practices for HRT prescription, considering possible effects on late‐life neurodegenerative disease.

